# Transcriptome Analysis of Key Genes Involved in the Initiation of Spermatogonial Stem Cell Differentiation

**DOI:** 10.3390/genes15020141

**Published:** 2024-01-23

**Authors:** Xinran Lu, Pengluo Yin, Huixia Li, Weijun Gao, Hua Jia, Wenzhi Ma

**Affiliations:** Key Laboratory of Fertility Preservation and Maintenance of Ministry of Education, Key Laboratory of Reproduction and Genetics of Ningxia Hui Autonomous Region, School of Basic Medical Science, Ningxia Medical University, Yinchuan 750004, China; 18835735160@163.com (X.L.); cindytrump0098@gmail.com (P.Y.); huixiali163@163.com (H.L.); gwj20190228@163.com (W.G.); 201100111@nxmu.edu.cn (H.J.)

**Keywords:** SSCs, transcriptome analysis, RNA-seq, differentiation

## Abstract

Purpose: The purpose of this study was to screen the genes and pathways that are involved in spermatogonia stem cell (SSC) differentiation regulation during the transition from A_undiff_ to A_1._ Methods: RNA sequencing was performed to screen differentially expressed genes at 1 d and 2 d after SSC differentiation culture. KEGG pathway enrichment and GO function analysis were performed to reveal the genes and pathways related to the initiation of early SSC differentiation. Results: The GO analysis showed that *Rpl21*, which regulates cell differentiation initiation, significantly increased after 1 day of SSC differentiation. The expressions of *Fn1*, *Cd9*, *Fgf2*, *Itgb1*, *Epha2*, *Ctgf*, *Cttn*, *Timp2* and *Fgfr1*, which are related to promoting differentiation, were up-regulated after 2 days of SSC differentiation. The analysis of the KEGG pathway revealed that RNA transport is the most enriched pathway 1 day after SSC differentiation. Hspa2, which promotes the differentiation of male reproductive cells, and Cdkn2a, which participates in the cell cycle, were significantly up-regulated. The p53 pathway and MAPK pathway were the most enriched pathways 2 days after SSC differentiation. *Cdkn1a*, *Hmga2*, *Thbs1* and *Cdkn2a*, microRNAs that promote cell differentiation, were also significantly up-regulated. Conclusions: RNA transport, the MAPK pathway and the p53 pathway may play vital roles in early SSC differentiation, and *Rpl21*, *Fn1*, *Cd9*, *Fgf2*, *Itgb1*, *Epha2*, *Ctgf*, *Cttn*, *Timp2*, *Fgfr1*, *Hspa2*, *Cdkn2a*, *Cdkn1a*, *Hmga2* and *Thbs1* are involved in the initiation of SSC differentiation. The findings of this study provide a reference for further revelations of the regulatory mechanism of SSC differentiation.

## 1. Introduction

Spermatogonial stem cells (SSCs), which are adult stem cells that can stably transmit genetic information to the next generation, are the foundation of spermatogenesis and male reproduction [1]. In mammalian spermatogenesis, the differentiation of SSCs into functional spermatozoa that fertilize the egg and ultimately produce offspring is a highly coordinated and dynamically evolving process [2]. Spermatogenesis is a continuous process that is divided into three main stages: mitosis of spermatogonia, meiosis of spermatocytes and metamorphosis of spermatocytes. The normal conduct of self-renewal and differentiation of SSCs can ensure the continuous production of large numbers of sperm throughout the reproductive life cycle, in which precise gene regulation is essential.

SSCs residing inside the basal lamina of the seminiferous tubules originate from primordial germ cells (PGCs). A_single_ (A_s_)-type SSCs are generally considered to be the least differentiated spermatogonocytes. Spermatogonia undergo incomplete cell division during differentiation. If an A_s_ spermatogonium divides into two and no intercellular bridges are produced, the division pattern is a symmetric self-renewal division, and they remain undifferentiated. Two A_s_ spermatogonia that produce intercellular bridges are referred to as A_pr_ spermatogonia and subsequently produce A_aligned-4_, A_aligned-8_ and A_aligned-16_ (A_al_). A_al_ spermatogonia undergo ulteriorly differentiation to form A_1_, A_2_, A_3_, A_4_, intermediate (Int) and B spermatogonia before entering meiosis [3]. A_s_, A_pr_, and A_al_ spermatogonia are called undifferentiated spermatogonia (A_undiff_), while A_1_, A_2_, A_3_, A_4_, intermediate (Int) and B spermatogonia are called differentiated spermatogonia (A_diff_). As spermatogonia are generally recognized as SSCs, and their number is extremely low in the entire adult testicular cell population, comprising only 0.03% of the total [4]. The ‘revised As model’ proposes that A_s_ spermatogonia maintain their numbers by undergoing complete cytokinesis [5]. During spermatogenesis, a subset of A_undiff_ becomes irreversible at a specific stage of the spermatogonium cycle, and differentiated spermatogonia cells are regulated by spermatogenic programs to undergo orderly mitosis [4,6]. In contrast, the ‘dynamic SSC model’, in which the fate of A_undiff_ cells is context-dependent and plastic, supports that A_undiff_ cells reversibly transition between differentiation-primed and self-renewing states based on the availability of niche-derived cues [7,8,9,10,11]. Due to the small number of SSCs, we cannot accurately identify the above two models, which has greatly hindered our understanding of SSC biology and the complexity of spermatogenesis.

Maintaining male fertility and maintaining a high enough level of spermatogenesis in the long term can be achieved only through a relative balance of various factors between different ecological niches, and these factors determine the fate of SSC development by promoting self-renewal, differentiation initiation or the spermatogenic commitment of undifferentiated spermatogonocytes (A_undiff_). To become sensitive to a differentiation-inducing stimulus (RA), A_undiff_ need to exit the self-renewing state and perform differentiation priming [12,13]. The activation of the mTORC1 pathway is essential for the self-renewal and differentiation of SSCs [14,15,16,17]. Exiting from the GFRa1 positive state requires cell size growth and the induction of a transcriptional program typical of differentiation-primed undifferentiated spermatogonia or progenitors [12,15,16]. The WNT/β-catenin signaling pathway plays a key role in the initiation of differentiation, facilitating A_undiff_’s transition from the self-renewing state upon perceiving the RA response [13,18,19,20]. It is generally considered that the induction of RAR in a subset of A_undiff_ gives cells the capacity to respond to RA [12,21,22]. RA is the inducer of differentiation in the germline, and to prevent a premature exit from the progenitor state that displays a latent self-renewal capacity, the enzyme CYP26B1, expressed by peritubulogenic myoid cells, degrades RA outside the tubule, preventing it from affecting the appropriate timing of spermatogenic initiation [23,24,25].

During the transition from A_al_ to A_1_ spermatogonia, the morphology and mitotic behavior of spermatogonial cells change irreversibly. This transformation is controlled by at least one external factor, the RA, and multiple intrinsic factors [26,27]. RA is required early on for the differentiation of the A_al_-to-A_1_ transition and then again for mature spermatid release. The RA-induced differentiation of spermatogonia occurs specifically during stages VII-VIII of the mouse seminiferous epithelial cycle [6]. Due to the effect of the RA, the A_undiff_ subpopulation of RAR-positive cells will transform into A_1_-differentiated spermatogonocytes, especially in stages VII-VIII, and at this time, they begin to express KIT, STRA 8 and other markers of spermatogenic differentiation [12,28,29,30]. Stra8 is a gene that is stimulated by RA to initiate meiosis. c-Kit expression is activated in mouse differentiated SSCs cultured in vitro [31]. SCF is produced by supporting cells and activates the phosphoinositoI3 kinase (PI3-K) signaling pathway by binding to c-Kit receptor tyrosine kinase to control cell growth, proliferation and differentiation. SCF was shown to improve the in vitro differentiation of SSCs by up-regulating differentiation genes (PRTM1, STRA8, c-KIT, PIWIL2) in OA rats [32]. The widely expressed growth factor activin A plays an important regulatory role in the fetus and testes, and its production and action are strictly controlled [33]. Activin A governs the development and proliferation of both germ and somatic cells during fetal life. In vitro studies have shown that activin A supports the differentiation of mouse and human SSCs [34,35]. Bone morphogenetic protein 4 (BMP4), belonging to the transforming growth factor-β(TGF-β) family, plays an essential role in spermatogenesis. BMP4 stimulates the differentiation of SSCs by up-regulating the transcription factor sohlh2 [36].

In recent years, research in this area has been committed to reproducing the entire process of spermatogenesis in vitro, which will hopefully present a solution to infertility in patients with azoospermia. The growth and development of SSCs, which serve as the basis for the entire process of spermatogenesis, are crucial aspects in reproducing this process. With the advancements in stem cell technology, the system of in vitro-induced differentiation culture has been further improved by adding growth factors or inducing a high expression of related genes, and then co-culturing with testicular somatic cells, among other methods [37,38,39]. Min Sun established a three-dimensional induction system to induce the differentiation of human SSCs into functional spermatozoa in vitro [40]. Peng Wang isolated SSCs from mouse testes and differentiated them into haploid male germ cells via RA induction [41]. However, the complex mechanism of the transition of SSCs to A_1_ spermatogonia is not fully understood, and the genes and pathways involved in the regulation of RA differentiation during this transition are still unknown. Therefore, in this study, we established an in vitro early differentiation culture system for SSCs and analyzed the key regulatory genes and signaling pathways during the transition of SSC to A_1_. The results of this study provide a reference for better understanding the pathogenesis of patients with transformation failure of A_undiff_ into A_diff_ spermatogonocytes. 

## 2. Materials and Methods

### 2.1. SSC Self-Renewal and Differentiation Culture

CD1^+^ SSCs were donated by Professor Ji Wu from Shanghai Jiao Tong University [42]. The medium for the self-renewal of SSCs was the same as used in the previous work of our lab. To be specific, SSCs were inoculated on feeder-layer Sandos inbred mouse (SIM)-derived 6-thioguanine- and ouabain-resistant (STO) embryonic fibroblast cells treated with mitomycin (M0503, Sigma, St Louis, MO, USA) [39]. The SSCs were cultured in Minimum Essential Medium α(MEM-α, 12571-063, Gibco, Grand Island, NY, USA) containing 10% fetal bovine serum (FBS) (16000-36, Gibco, Grand Island, NY, USA), 2mM glutamine (G7012, Sigma, St Louis, MO, USA), 1× nonessential amino acid (NEAA, 11140-050, Gibco, Grand Island, NY, USA) solution, 0.5× pen/strep (15240-062, Invitrogen, Grand Island, NY, USA), 1× β-mercaptoethanol (β-ME, M3148, Sigma, St Louis, MO, USA), 100 μg/mL transferrin (T1428, Sigma, MO, USA), 25 μg/mL insulin (I1882, Sigma, St Louis, MO, USA), 60 ng/mL progesterone (P8783, Sigma, MO, USA), 60 μM putrescine (P5780, Sigma, St Louis, MO, USA) and 8 ng/mL basic fibroblast growth factor (bFGF, F0291, Sigma, St Louis, MO, USA) and grown in a humidified atmosphere in an incubator with 5% CO_2_ at 37 °C. For the differentiation culture, stem cells were cultured in differentiation medium at a constant 34 °C with 5% CO_2_ [43]. The differentiation culture medium was prepared by adding differentiation factor SCF (100 ng/mL, R&D Systems, Minnneapolis, MN, USA), BMP4 (20 ng/mL, R&D Systems, Minnneapolis, MN, USA), RA (10^−6^ M, Sigma, MO, USA) and activin A (100 ng/mL, R&D Systems, Minnneapolis, MN, USA) on the basis of the self-renewal culture medium [44].

In order to simulate the optimal temperature for sperm generation in vivo, we cultured SSCs in an incubator at 34 °C. Factors that promote SSC differentiation, namely SCF, BMP4, RA and activin A, were added into the differentiation medium. Previous studies have shown that Stra8 expression increases significantly 24 h after differentiation induced by RA and BMP4 [45,46]. Thus, we selected SSCs after 1 d and 2 d of induction for RNA sequencing analysis.

### 2.2. Quantitative Real-Time PCR

Quantitative real-time PCR (qPCR) was used to detect the differentiation genes of SSCs. The primers were synthesized by Sango biotech (Shanghai, China) Co., Ltd.: GAPDH, Forward: AACGGATTTGGCCGTATTGG, Reverse: CATTCTCGGCCTTGACTGTG; ID4, Forward: TGCAGTGCGATATGAACGAC, Reverse: GCAGGATCTCCACTTTGCTG; Thy-1, Forward: GCTCTCCTGCTCTCAGTCTT, Reverse: GCTGAACTCATGCTGGATGG; c-kit, Forward: GGGACACATTTACGGTGGTG, Reverse: GCTTTACCTGGGCTATGTGC; Stra8, Forward: TTGACGTGGCAAGTTTCCTG, Reverse: GGGCTCTGGTTCCTGGT TTA; Rec8, Forward: CCCGCTTCTCCCTCTATCTC, Reverse: CGATGTAGGT GCTCCAGGAT; Sycp3, Forward: CCAATCAGCAGAGAGCTTGG, Reverse: CC TCGAAGCATCTGAGGAAA; Ovol1, Forward: TGTCTTACAGGCAGAGCACA, Reverse: GGCCTGTCTCTGTAAGTGGT. Tip Green qPCR SuperMix (Q311-02, Vazyme Biotech, Nanjing, China) was used to detect the differentiated genes in accordance with the instructions. The 2^−ΔΔCT^ methods were adopted for data analysis.

### 2.3. RNA Sequencing and GO and KEGG Enrichment Analyses

Illumina sequencing was performed on different libraries after qualified library inspection. The basic principle of sequencing was Sequencing by Synthesis. Four fluorescently labeled dNTPs, DNA polymerase and adaptor primers were added to the sequenced flow cell for amplification. When cDNA is used as a template and primers and dNTPs are used for PCR amplification, fluorescent dyes or probes are added to the reaction system. These fluorescent molecules can be combined with the amplified sequence, and the fluorescence signal intensity can then be monitored in real time using a fluorescence quantitative PCR instrument (qPCR). The light signal is converted into sequencing peaks using computer software. The sequence information of the fragment to be tested was thus obtained.

ClusterProfiler software was used for GO functional enrichment analysis and KEGG pathway enrichment analysis. All enriched differentially expressed genes (DEGs) were mapped to terms in the GO database and KEGG database.

### 2.4. Statistics

Data were recorded in the form of mean ± standard error of the mean. One-way ANOVA was utilized to probe the discrepancies between different groups, and all analyses with a *p*-value < 0.05 or 0.01 were deemed to be significant.

## 3. Results

### 3.1. Establishment of SSCs in In Vitro Differentiation Culture System

**Figure 1:**
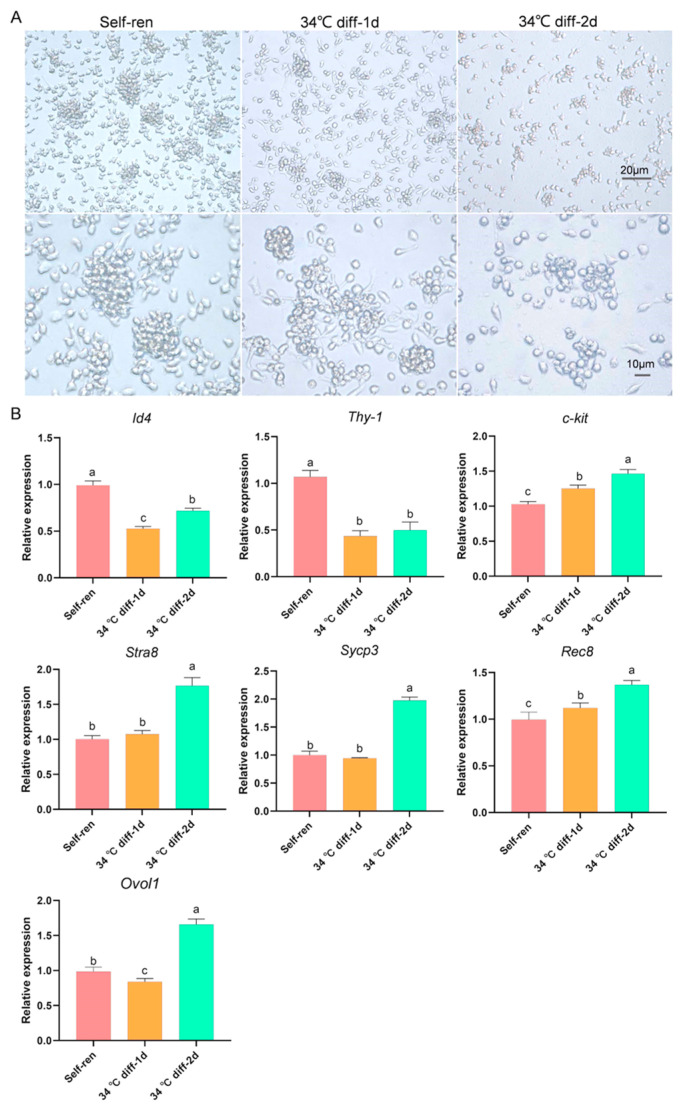
Establishment of SSCs in an in vitro differentiation culture system. (**A**). SSCs grew well in the 37 °C self-renewal culture group and in the 34 °C differentiation culture group. (**B**). The expressions of undifferentiated spermatogonial marker genes *Id4* and *Thy-1*, differentiated spermatogonial marker gene *c-kit*, and meiosis-related genes *Stra8*, *Rec8* and *Sycp3* after differentiation and culture. Self-ren, self-renewal; 34 °C diff, differentiation culture at 34 °C; 34 °C diff-1d, day one of differentiation culture at 34 °C; 34 °C diff-2d, day two of differentiation culture at 34 °C. a, b, c, different letters indicate a significant difference (*p* < 0.05).

In order to investigate the transcriptomic differences of spermatogonial stem cells during the initiation of their differentiation, we established an in vitro SSC differentiation culture system to avoid the limitations of the small number and asynchronous development of spermatogonial stem cells in vivo. The results show that the differentiated SSCs cultured at 34 °C showed obvious colony-like growth on the second day of culture in the differentiation media supplemented with SCF, BMP4, RA and activin A (Figure 1A). The expression of undifferentiated spermatogonial marker genes *Id4* and *Thy-1* was inhibited from the first day after differentiation culture, and the expression of spermatogonial differentiation marker gene *c-kit* was up-regulated. Except for *Rec8*, the expression of genes involved in meiosis, namely *Stra8, Sycp3* and *Ovol1* showed no significant change on the first day of differentiation, but the expressions of all indicators were significantly up-regulated on the second day of differentiation culture. (Figure 1B). Together, the above results verify that the SSC differentiation system was successfully established.

### 3.2. Analysis of Differentially Expressed Genes after Differentiation Culture

**Figure 2:**
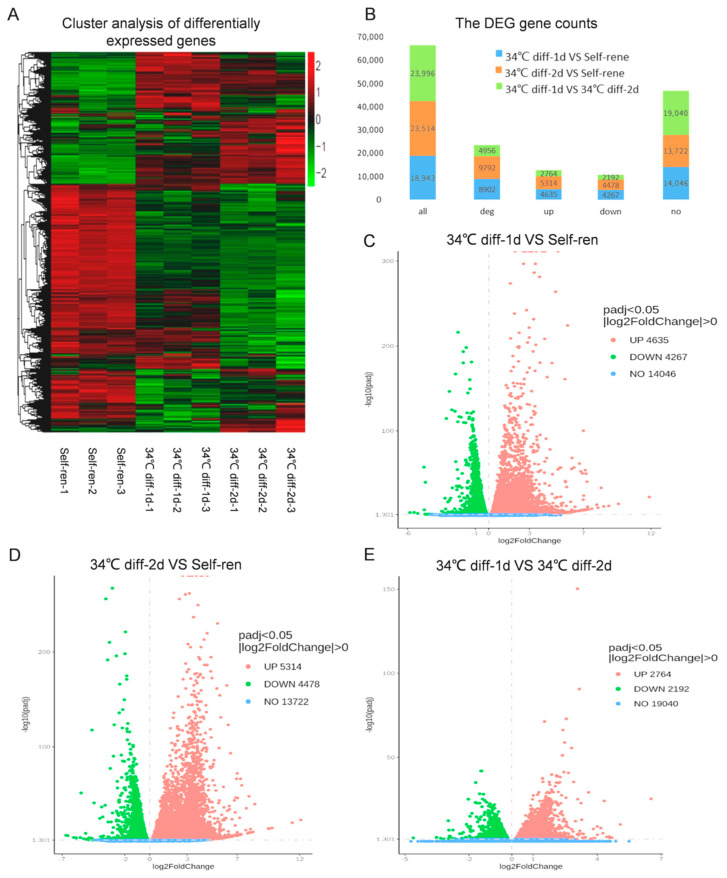
Analysis of differentially expressed genes during SSC differentiation. (**A**). Heatmap of differentially expressed genes among the self-continuation group, 34 °C diff−1d group and 34 °C diff−2d group. Red stripes represent highly expressed genes, while green stripes represent low-expression genes. (**B**). The total number of genes and differentially expressed genes (DEGs) identified from self-ren group vs 34 °C diff−1d group, self-ren group vs 34 °C diff−2d group and 34 °C diff−1d group vs. 34 °C diff−2d group. (**C**). Volcano map of DEGs between the self-renewal and 34 °C diff−1d groups. (**D**). Volcano map of DEGs between the self-renewal and 34 °C diff−2d groups. (**E**). Volcano map of DEGs between the 34 °C diff−1d and 34 °C diff−2d groups. The x-axis is the log2 scale of the fold change of gene expression in the self-renewal and differentiation groups (log2(fold change)). Negative values indicate down-regulation; positive values indicate up-regulation. The y-axis is the minus log10 scale of the adjusted *p* values (elog10 (padj)), which indicate the significance levels of expression difference. The red dots represent significantly up-regulated genes with at least a two-fold change, while the green dots represent significantly down-regulated genes with at least a two-fold change.

To investigate transcriptome expression changes after initiation of SSC differentiation, RNA-Seq analysis was performed on the SSCs 1 and 2 days after in vitro differentiation. Cluster analysis was conducted on the differential gene set (Figure 2A, where the red represents highly expressed genes, and the green represents a relatively low expression of genes). The expression patterns of the three repeated samples in the self-continuation group, 34 °C diff-1d group and 34 °C diff-2d group were similar, and the expression differences were obvious among the self-continuation group, 34 °C diff-1d group and 34 °C diff-2d group (Figure 2A). The total number of genes identified in the self-ren group vs. 34 °C diff-1d group, self-ren group vs. 34 °C diff-2d group and 34 °C diff-1d group vs. 34 °C diff-2d group were 18,943, 23,514 and 23,996, respectively (Figure 2B). Compared with the self-renewal group, we found that the expression of 8902 genes changed significantly (two-fold difference between two stages with an FDR less than 0.05) after 1 day of differentiation culture, among which, 4635 were significantly up-regulated and 4267 were down-regulated in the 37 °C self-renewal group (Figure 2B,C). Moreover, 9792 genes had significantly changed expressions (two-fold difference between two stages with an FDR less than 0.05) after 2 days of differentiation culture, among which, 5314 were significantly up-regulated and 4478 were down-regulated in the self-ren group (Figure 2B,D). In the 34 °C diff-1d group, 4956 gene expressions changed significantly (two-fold difference between two stages with an FDR less than 0.05) after 2 days of differentiation culture, among which, 2764 were significantly up-regulated and 21,192 were down-regulated in the self-ren group (Figure 2B,E).

### 3.3. Gene Ontology (GO) Analysis of the Differentially Expressed Genes

The dynamic processes of the DEGs obtained from RNA-Seq during SSC differentiation were analyzed via GO from three aspects: biological process (BP), cell component (CC) and molecular function (MF). 

**Figure 3:**
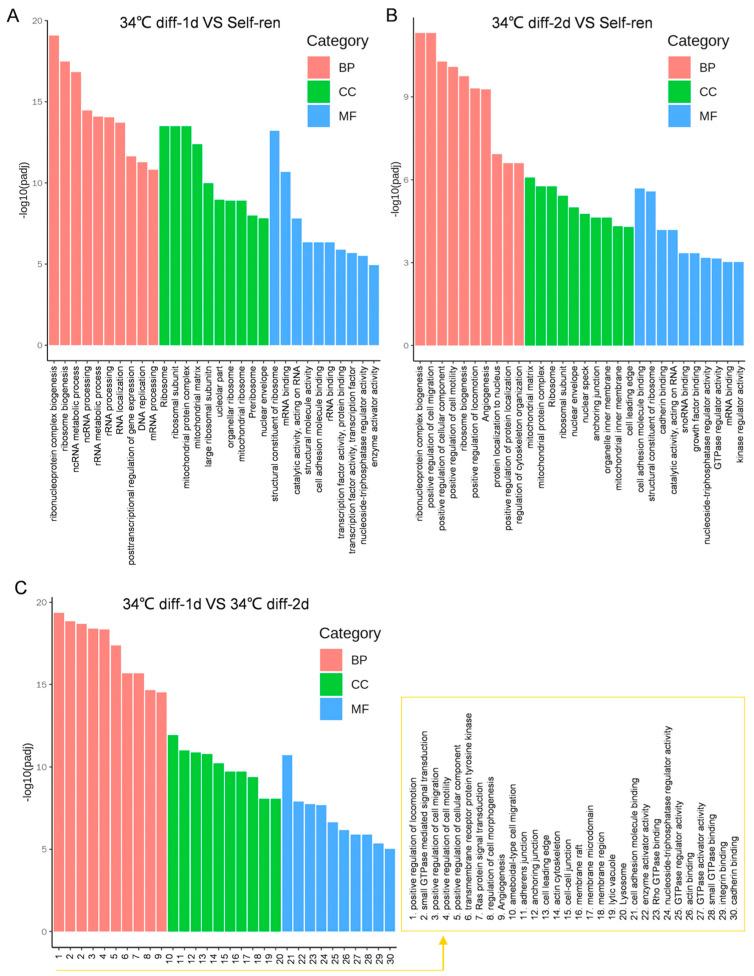
The GO term is enriched between the self-renewal and differentiation groups. The abscissa is the GO term, and the ordinate represents the GO term significance level in the GO enrichment analysis. Higher values mean greater significance. The red stripes represent the BP subset, the green stripes represent the CC subset, and the blue stripes represent the MF subset. (**A**). The top 30 GO terms in the enrichment analysis of the self-renewal group and on the first day of differentiation. (**B**). The top 30 GO terms in the enrichment analysis of the self-renewal group and on the second day of differentiation. (**C**). The top 30 GO terms in the enrichment analysis of the first-day group and on the second day of differentiation.

**Figure 4:**
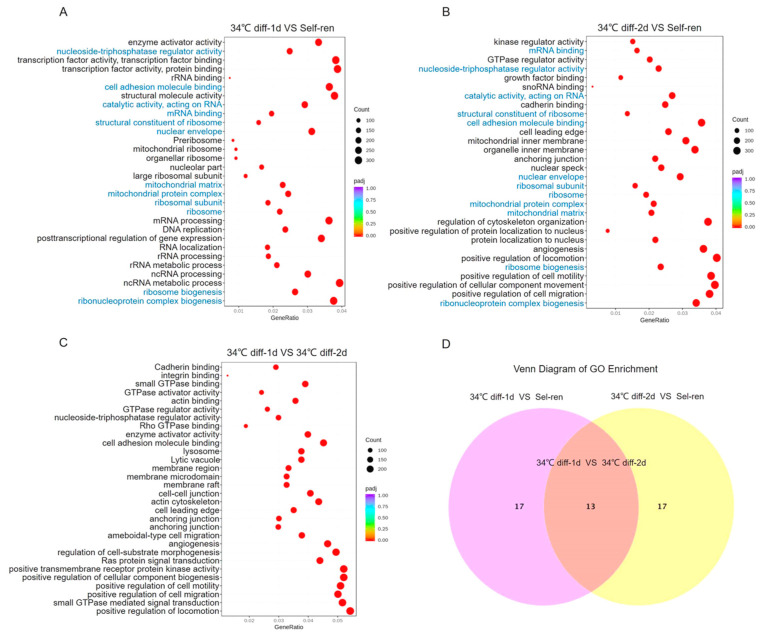
GO analysis of the differentially expressed genes. (**A**). The top 30 enriched GO terms on the first day of differentiation compared with self-renewal. (**B**). The 30 enriched GO terms on the second day of differentiation compared with self-renewal. (**C**). Venn diagram of GO terms in the 34 °C differentiation after 1 d vs. self-ren groups and 34 °C differentiation after 2 d vs. self-ren groups. Twelve GO terms were co-enriched. (**D**). The top 30 enriched GO terms on the second day of differentiation compared with the first day of differentiation. The abscissa is the ratio of the number of differential genes enriched on the GO terms to the totality, and the ordinate is the GO term. The size of the dot indicates the number of genes enriched on the GO term. The degree of enrichment from small to large is represented by the color from purple to red.

Compared with self-renewing SSCs, the BP items with the highest enrichment after 1 day of SSC differentiation included ribonucleoprotein complex biogenesis, ribosome biogenesis and ncRNA metabolic processes (Figure 3A and Figure 4A). Some up-regulated genes participated in the increase in metabolism, including *Cdkn2a*, *Mrpl10*, *Rnasel*, *Rpsa* and *Ago4* (Table 1). Ribosomes and ribosome subunits are enriched in CC terms (Figure 3A and Figure 4A). The expressions of *Rbm3*, *Lcmt2* and *Hsd17b10* genes related to protein synthesis are significantly up-regulated (Table 1). The most enriched MF terms are structural constituents of ribosome and mRNA binding (Figure 3A and Figure 4A). *Rpl21*, which regulates cell differentiation initiation, significantly increased (Table 1).

Compared with self-renewing SSCs, the BP terms with the highest enrichment after 2 days of SSC differentiation were ribonucleoprotein complex biogenesis and the positive regulation of cell migration (Figure 3B and Figure 4B). The *Anxa1* gene, which promotes differentiation, and the *Eif2a* gene, which promotes cell development, were significantly up-regulated (Table 2). The mitochondrial matrix is mainly enriched in the CC term (Figure 3B and Figure 4B). Cell adhesion molecule binding was enriched in the MF term (Figure 3B and Figure 4B). The expressions of *Fn1*, *Cttn*, *Timp2* and *Fgfr1,* which are related to promoting differentiation, were up-regulated. The Venn diagram in Figure 4C shows 12 signaling pathways co-enriched on the first and second day of SSC differentiation, including ribonucleoprotein complex biogenesis and ribosome biogenesis, etc.

A GO enrichment analysis of the DEGs was performed on the second day of SSC differentiation, and the results were compared with those from the first day of differentiation, and the top 30 GO pathways were screened out (Figure 3C and Figure 4D). The results showed that BP terminology mainly focused on the positive regulation of motor, small GTPase-mediated signal transduction and the positive regulation of cell migration. Adhesion junction was mainly enriched in the CC terms. Genes related to promoting differentiation, including *Jag1*, *Afap1*, *Itgb1*, *Itgav*, *Tns3*, etc. were significantly up-regulated (Table 3). The most enriched MF term was cell adhesion molecule binding. Genes related to promoting differentiation, including *Fn1*, *Cd9*, *Fgf2*, *Itgb1*, *Epha2*, *Ctgf*, etc., were also significantly up-regulated (Table 3).

### 3.4. Kyoto Encyclopedia of Genes and Genomes (KEGG) Analysis of the Differentially Expressed Genes

**Figure 5:**
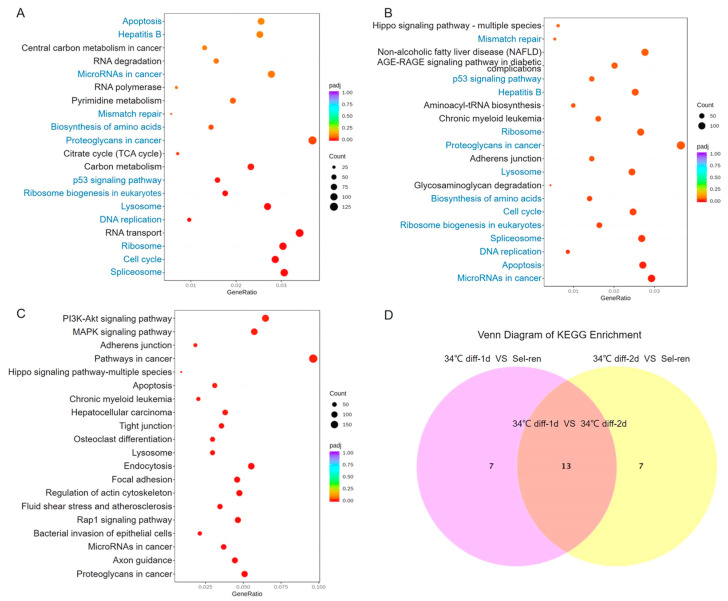
KEGG analysis of the differentially expressed genes. (**A**). The top 20 enriched KEGG pathway on the first day of differentiation compared with self-renewal. (**B**). On day 2 of differentiation, the top 20 KEGG pathways enriched compared to the self-renewal group. (**C**). Venn diagram of KEGG pathway in the 34 °C differentiation-1d group vs. self-ren group and 34 °C differentiation-2d group vs. self-ren group. Thirteen KEGG signaling pathways were co-enriched. (**D**). The top 20 enriched KEGG pathway on the second day of differentiation compared with the first day of differentiation. The abscissa is the ratio of the number of differential genes enriched on the KEGG pathway terms to the totality, and the ordinate is the functional pathway. The signaling pathways labeled in blue were enriched on both the first and second day of SSC differentiation.

The KEGG database was used to evaluate the enrichment of the DEGs in order to identify the most significantly enriched pathways and then determine whether genes related to SSC differentiation participate in those specific pathways. Compared with the self-renewal culture, the top 20 KEGG pathways after 1 d of induced differentiation culture are shown in Figure 5A. According to the enrichment results, ribosome, cell cycle, spliceosome, apoptosis and RNA transport are the most enriched pathways. Among them, *Ncbp1*, which mediates RNA in the spliceosome pathway; *Hspa2*, which promotes the differentiation of male reproductive cells; and *Cdkn2a*, which participates in the cell cycle, were significantly up-regulated (Table 4), indicating strong cell metabolism. 

The 2 d of induced differentiation culture group was enriched for the top 20 KEGG pathways compared to the self-renewal group, as shown in Figure 5B. The study revealed that the apoptosis, spliceosome and p53 pathways were the most enriched pathways. Among them, the expressions of *Cdkn1a*, *Hmga2*, *Thbs1* and *Cdkn2a* in MicroRNAs that promote cell differentiation in cancer pathways increased (Table 5), indicating that the differentiation of SSCs is dominant. 

The Venn diagram in Figure 5D shows 13 signaling pathways co-enriched on the first and second day of induced differentiation, including the p53 signaling pathway, spliceosome pathway, etc. The expressions of downstream genes *Thbs1*, *Rrm2b*, *Ccnd1*, *Cdkn1a* and *Mdm2* of the p53 signaling pathway were up-regulated. KEGG enrichment analysis was performed on differentially expressed genes on the second day of differentiation taking the first day of differentiation as the control, and the top 20 KEGG pathways were screened out (Figure 5C). The PI3K-Akt pathway, MAPK signaling pathway and proteoglycans in cancer were the most enriched pathways. Among them, *Map2k1*, *Kras*, *Pak1*, *Akt3* and *Fgf2* were up-regulated (Table 6), and *Map2k1*, *Kras*, *Pak1*, *Akt3* and *Fgf2* were up-regulated (Table 6), suggesting that the MAPK signaling pathway may regulate SSC differentiation.

## 4. Discussion

SSCs account for just 0.02–0.03% of all testicular germ cells, and SSCs in testicular tissues are affected by many factors, such as Sertoli cells, spermatogenic cells at all levels and the testicular microenvironment [47]. The differentiation of SSCs is an extremely complex process involving the interaction of multiple genes and the regulation of signaling pathways. It is difficult to study spermatogonial stem cell differentiation in vivo. Therefore, we used in vitro-cultured SSCs to find the genes and pathways involved in the regulation of RA differentiation during the transition from A_undiff_ to A_1_. 

The cell morphology was observed on the first and second days to be progressing in a colony-like manner in this study, which is consistent with the previously reported morphology of stem cells after induced differentiation [48]. Subsequently, we found that the expressions of the SSC-specific marker genes *Thy-1* and *Id4* decreased, while the differentiated spermatogonial cell-specific marker gene *c-kit*, meiosis-initiating gene *Stra8*, meiosis-specific gene *Rec8* and primary spermatocyte-specific marker gene *Sycp3* were all up-regulated, and the effect was more pronounced on the second day of induced differentiation. Above all, these results indicate that the induced SSC differentiation system was established successfully. SSCs have a key gene, SCF, which contributes to the homeostasis of self-renewal and differentiation and plays a key role in binding with c-kit [49]. Activin A is essential for the growth and maturation of spermatogonial stem cells [50], and activin A is a cytokine widely used in the differentiation of stem cells in vitro [51,52,53]. We can further study the differentiation process of SSCs in vitro by using the above culture system. In this study, we compared and analyzed RNA-seq data from the self-renewal group and the induced differentiation system on day 1 and day 2 to understand the regulatory mechanism of SSC differentiation initiation. RNA-seq, a high-throughput sequencing technology, can be applied to investigate, characterize and quantify the transcriptome. Transcriptome sequencing technology is widely used in basic medical research, clinical diagnosis and drug development because of its ability to quickly and comprehensively detect specific cells in the specific tissues of a certain species [54]. RNA-seq technology can accurately detect the mRNA involved in the initiation of SSC differentiation. The GO enrichment analysis in our study found that differentiation-promoting genes *Jag1*, *Afap1*, *Itgb1*, *Itgav*, *Tns3*, *Fn1*, *Cd9*, *Fgf2*, *Itgb1*, *Epha2* and *Ctgf* were significantly up-regulated. Notch’s ligand Jag1 has been reported to possibly stimulate the differentiation of stem cells [55]. When human mesenchymal stem cells differentiate into cartilage, Itgb1 activates the ERK signaling pathway [56]. Tns3 mediates the excitation of ITGβ1 to irritate the differentiation of MSCs [57]. Fn1 facilitates the differentiation of osteocyte by activating the TGF-β/PI3K/Akt pathway [58]. Cd9 mediates the activation of the PI3K/Akt pathway to accelerate the differentiation of keratinocyte [59]. In summary, these genes are essential for the differentiation of spermatogonial stem cells, suggesting that we should pay close attention to their regulatory role in the differentiation of SSCs.

KEGG analysis showed that the p53 pathway and MAPK pathway were the most enriched pathways 2 days after SSC differentiation. The MAPK pathway is integral for eukaryotic cell growth and proliferation by transducing extracellular signals into the inside, causing intracellular responses [45]. At present, the MAPK pathway has been found to be reflected in a variety of cell differentiation processes. Lian-mei Zhao [46] demonstrated that the activation of the p38-MAPK pathway can promote the differentiation of melanoma cells. Peng Zhang [49] found that the differentiation of bone marrow mesenchymal stem cells is mediated by the p38-MAPK pathway. Yeon-Jeong Jang [50] found that the MAPK and ERK/JNK pathways inhibit the differentiation of adipocytes by regulating the expression of PPAR+ and C/EBP. Tilo Kunath [51] found that the Ras-Mek-Erk pathway, a downstream signaling pathway of MAPK, plays an essential role in the process of differentiation of mouse embryonic stem cells. We investigated whether the MAPK signaling pathway is involved in the regulation of the differentiation of SSCs cultured in vitro. Transcriptome sequencing revealed that the expression of Rbm3, which is associated with protein synthesis, was significantly up-regulated 1 day after SSC differentiation compared to self-renewing SSCs. RNA-binding motif protein 3 (RBM3) is a specific cold shock protein, a member of the RNA-binding protein family, and is rapidly up-regulated at low temperatures and low oxygen [60,61]. It has been shown that the expression of RBM 3 is essential for the orderly progression of some cell cycles and the onset of mitosis [62]. RBM3 is able to stimulate osteoblast differentiation through the ERK signaling pathway, and it also regulates the expression of mitogen-activated protein kinase (MAPK) [63]. In addition, we found that *Vegfa*, *Akt3*, *Fgf2*, *Met*, *Egfr* and *Igf1r* were significantly up-regulated in the MAPK pathway of differentiated SSCs induced after 2 d compared with after 1 d through transcriptome sequencing. MAP2K1, the ERK kinase gene responsible for encoding ERK kinase activation, is an essential component of MAP kinase transduction [53]. Studies show that MAP2K1 is vital for the development of embryonic ectoderm. Vickram Bissonauth [52] reported that MAP2K1 deficiency can lead to mouse embryonic death. MAP2K1ip1 is a MAP2K1 interacting protein that promotes the differentiation of embryonic stem cells through the activation of the Ras-Mek-Erk pathway [64]. However, the mechanism behind promoting the differentiation of SSCs needs further study. As early as 2011, it was discovered that vascular endothelial growth factor A (*Vegfa*) can promote the differentiation of SSCs cultured in vitro [65], which is consistent with our sequencing results. One of the signaling pathways of human embryonic stem cell differentiation is facilitated by FGF2 through the activation of the MEK-ERK pathway [66]. In our sequencing results, the expression of *Fgf2* was notably up-regulated, so we speculate it may have a vital function in promoting the differentiation of SSCs.

RNA sequencing demonstrated that the p53 pathway was enriched in differentiated SSCs induced after 1 d and 2 d. It was reported that p53 regulates self-renewal, differentiation, autophagy and other normal life activities [67]. Studies show that p53 promotes the differentiation of human embryonic stem cells by regulating the cell cycle and microRNA and can stimulate mouse embryonic stem cells to differentiate into embryoid bodies [68,69]. During the differentiation of normal erythrocyte, p53 promotes differentiation through ribosomal biogenesis [70]. By sequencing differentiated SSCs induced after 1 d and 2 d, we observed that the expressions of *Thbs1*, *Rrm2b*, *Ccnd1*, *Cdkn1a*, *Mdm2*, etc. of p53 downstream molecules were up-regulated.

MDM2, the strongest p53-negative regulator discovered so far, can bind to the p53 protein and exert corresponding biological regulation [71]. Many studies show that MDM2 promotes the self-renewal and differentiation of mouse and human airway epithelial basal stem cells by regulating p53 protein and induces cell cycle arrest and apoptosis [72], which implies that MDM2 may regulate the differentiation and self-renewal of SSCs through the activated p53 pathway. The CDKN1a (cyclin-dependent kinase inhibitor 1a)-encoding p21 protein executes a negative regulatory function on the cell cycle by stopping the cell cycle in the G1 phase [73]. As an important downstream target gene of p53, p21 is actively involved in the growth and development of spermatogonial stem cells. Studies indicate that in acute myeloid leukemia, lncRNA HOTAIR promotes myeloid differentiation by up-regulating p21 [74]. However, whether CDKN1a promotes the differentiation of SSCs requires further verification. CCND1 is a cyclin that mainly regulates the cell cycle transition from the pre-DNA synthesis phase (G1 phase) to the DNA synthesis phase (S phase), promoting cell proliferation and differentiation [75]. The abnormal expression of CCND1 is related to the occurrence and development of various tumors [76]. Studies show that inhibiting autophagy by silencing CCND1 in turn inhibits the differentiation of liver cancer [77]. However, whether CCND1 promotes the differentiation of SSCs through autophagy requires further research.

Male infertility has always been an important issue in the field of reproductive health research. Obtaining mature and functional sperm through in vitro culture is the key to achieving effective treatment, especially for azoospermia patients. SSCs are crucial as the basis for the entire spermatogenesis process. Therefore, in this study, we constructed an early in vitro differentiation system for SSCs, and identified some key regulators and signaling pathways for self-renewal and differentiation during the transition from SSCs to A_1_ spermatogonial cells using RNA-seq analysis. This will provide a new perspective for patients whose A_udiff_ to A_diff_ spermatogonial differentiation has failed.

## 5. Conclusions

The RNA transport, MAPK pathway and p53 pathway may play vital roles in early SSC differentiation, and *Rpl21*, *Fn1*, *Cd9*, *Fgf2*, *Itgb1*, *Epha2*, *Ctgf*, *Cttn*, *Timp2*, *Fgfr1*, *Hspa2*, *Cdkn2a*, *Cdkn1a*, *Hmga2* and *Thbs1* are involved in the initiation of SSC differentiation, which provide a reference for further revelations of the regulatory mechanism of SSC differentiation.

## Figures and Tables

**Table 1:** Significantly enriched GO terms and DEGs in SSCs on the first day of differentiation compared with self-renewal.

GO-ID	GO Term	UP-Genes	DOWN-Genes	*p*-Value	Type
GO:0022613	ribonucleoprotein complexbiogenesis	*Cdkn2a*, *Mrpl10*	*Gemin5*, *Nhp2*, *Mrto4*, *Rpl3*, *Rpl12*, *Rps23*	1.48 × 10^−23^	BP
GO:0042254	Ribosome biogenesis	*Rnasel*, *Rpsa*	*Rps27l*, *Surf6*, *Mrpl20*, *Rps2*, *Mrps7*, *Rpl7l1*, *Rsl24d1*	1.17 × 10^−21^	BP
GO:0034660	ncRNA metabolic process	*Ago4*	*Snu13*, *Npm1*, *Rpl11*, *Rpl35a*, *Mrpl44*	7.85 × 10^−21^	BP
GO:0034470	ncRNA processing	*Nsun3*, *Riok3*	*Rps24*, *Rpl10a*, *Rps7*, *Rpl14*, *Rpl7*	2.40 × 10^−18^	BP
GO:0016072	rRNA metabolic process	*Nsun4*, *Wdr37*	*Mrps11*, *Rpl5*, *Rps21*, *Rps6*, *Rps16*	7.24 × 10^−18^	BP
GO:0006364	rRNA processing	*Tsr3*	*Mrps9*, *Rps19*, *Rps17*, *Rpl26*	9.46 × 10^−18^	BP
GO:0003735	structural constituent of ribosome	*Rpl17*, *Rpl21*	*Mrpl11*, *Rps15*, *Rps14*, *Rps8*, *Rpl27*, *Rpl34*	5.78 × 10^−17^	MF
GO:0005840	ribosome	*Rbm3*	*Rpl10*, *Rps25*, *Rpl38*, *Rpl35*, *Rsl1d1*	9.91 × 10^−17^	CC
GO:0044391	ribosomal subunit	*Lcmt2*, *Hsd17b10*	*Mrpl1*, *Imp3*, *Rpl6*, *Rps5*, *Rps18*, *Rpl23a*, *Rpl13a*, *Ddx3x*	9.91 × 10^−17^	CC

**Table 2:** Significantly enriched GO terms and DEGs in SSCs on the second day of differentiation compared with self-renewal.

GO-ID	GO Term	UP-Genes	DOWN-Genes	*p*-Value	Type
GO:0042254	ribosome biogenesis	*Cdkn2a*, *Riok3*, *Rnasel*	*Npm1*, *C1qbp*, *Nop53*	1.54 × 10^−13^	BP
GO:0022613	ribonucleoprotein complex biogenesis	*Eif2a*, *Nmd3*	*Hsp90ab1*, *Dicer1*	1.21 × 10^−15^	BP
GO:0030335	positive regulation of cell migration	*Fn1*, *Fgfr1*, *Thbs1*, *Anxa1*	*Tert*, *Mapk14*, *Akt1*	1.71 × 10^−15^	BP
GO:0001667	ameboidal-type cell migration	*Epha2*, *Ilk*, *Jup*	*Pdcd6*, *Itgb3*, *Prkd2*	8.48 × 10^−10^	BP
GO:0034504	Protein localization to nucleus	*Lrrk2*, *Ptgs2*, *Lif*, *BMP4*, *Cd2ap*	*Cdk5rap3*, *Tmem173*, *Park7*	1.65 × 10^−10^	BP
GO:1900182	positive regulation of protein localization to nucleus	*Smo*, *Src*, *Cdkn2a*	*Jak2*, *Sesn2*, *Hyal2*, *Stk11*	4.64 × 10^−10^	BP
GO:0010608	posttranscriptional regulation of gene expression	*Ep300*, *Parp9*	*Tgfb1*, *Mapk1*, *Tpr*	8.42 × 10^−10^	BP
GO:0050839	cell adhesion molecule binding	*Fn1*, *Cttn*, *Timp2*, *Fgfr1*	*Pdlim1*, *Twf2*	2.06 × 10^−6^	MF
GO:0051098	regulation of binding	*Jak2*, *Nmd3 Tgfb2*	*Parp1*	4.78 × 10^−10^	BP
GO:0001525	angiogenesis	*Card10*, *Ngfr*, *Eif2ak3*, *Tek*	*Gpld1*	5.36 × 10^−10^	BP

**Table 3:** Significantly enriched GO terms and DEGs in SSCs on the second day of differentiation compared with the first day of differentiation.

GO-ID	GO Term	UP-Genes	*p*-Value	Type
GO:0001525	angiogenesis	*Ptgs2*, *Plau*	4.05 × 10^−30^	BP
GO:0040017	positive regulation of locomotion	*Prl2c2*, *Hbegf*	2.67 × 10^−28^	BP
GO:0001667	ameboidal-type cell migration	*Grem1*, *Vegfa*, *Ccbe1*	2.76 × 10^−27^	BP
GO:0030335	positive regulation of cell migration	*Spry2*, *Jun*, *Sphk1*	5.15 × 10^−27^	BP
GO:2000147	positive regulation of cell motility	*Fn1*, *Thbs1*	6.66 × 10^−27^	BP
GO:0071363	cellular response to growth factor stimulus	*Adgra2*, *Ccl2*	8.38 × 10^−27^	BP
GO:0051272	positive regulation of cellular component movement	*Amotl1*, *Itgb1*	1.10 × 10^−26^	BP
GO:0070848	response to growth factor	*Ets1*, *Fgf2*	1.38 × 10^−26^	BP
GO:0005912	adherens junction	*Jag1*, *Afap1*, *Itgb1*, *Itgav Tns3*	1.12 × 10^−19^	CC
GO:0050839	cell adhesion molecule binding	*Fn1*, *Cd9*, *Fgf2*, *Itgb1*, *Epha2*, *Ctgf*	2.88 × 10^−16^	MF

**Table 4:** Significantly enriched KEGG pathway and DEGs in SSCs on the first day of differentiation compared with self-renewal.

KWGG-ID	KEGG Pathway	UP-Genes	DOWN-Genes	*p*-Value
mmu03040	Spliceosome	*Ncbp1*, *Hspa2*, *Hspa1a*	*Ddx39b*, *Ncbp2*, *Alyref*, *Snu13*	2.65 × 10^−9^
mmu04110	Cell cycle	*Cdkn2a*, *Gadd45b*	*Ccne1*, *Ccnd2*, *Ccnb1*, *Cdk1*	4.87 × 10^−9^
mmu03010	Ribosome	*Mrpl10*, *Rpsa*, *Rpl21*, *Rpl17*, *Rpl34-ps1*	*Mrpl12*, *Rpl35a*	1.06 × 10^−7^
mmu03013	RNA transport	*Magohb*, *Lig1*, *Nxt2*	*Pop5*, *Pop4*, *Pop1*, *Nxt1*, *Nxf1*, *Nvl*	1.53 × 10^−7^
mmu03030	DNA replication	*Rfc2*, *Pold4*	*Pola1*, *Pole4*, *Prim2*, *Pola2*, *Pole*	3.50 × 10^−7^
mmu04142	Lysosome	*Smo*, *Src*, *Cdkn2a*	*Manba*, *Atp6v1h*, *Gga2*, *Ap1g2*	2.12 × 10^−6^
mmu03008	Ribosome biogenesis in eukaryotes	*Xrn1*, *Nxt2*	*Ran*, *Rpp25*, *Xpo1*	6.79 × 10^−6^
mmu04115	p53 signaling pathway	*Thbs1*, *Rrm2b*, *Ccnd1*, *Mdm2*, *Fas*	*Mcm5*, *Mcm2*, *Mcm6*, *Bub1b*, *Cdk4*, *Cdk6*, *Bax*, *Cdk2*	5.27 × 10^−5^
mmu01200	Carbon metabolism	*Idh1*, *Ogdhl*	*Shmt2*, *Psat1*, *Pfkl*, *Eno1b*	8.96 × 10^−5^
mmu05205	Proteoglycans in cancer	*Mdm2*, *Ctsl*, *Cd63*	*Casp3*, *Myc*	6.44 × 10^−4^

**Table 5:** Significantly enriched KEGG pathway and DEGs in SSCs on the second day of differentiation compared with self-renewal.

KWGG-ID	KEGG Pathway	UP-Genes	DOWN-Genes	*p*-Value
mmu05206	MicroRNAs in cancer	*Cdkn1a*, *Hmga2*, *Thbs1*, *Cdkn2a*	*E2f2*, *Ccne1*	1.06 × 10^−5^
mmu04210	Apoptosis	*Ctsd*, *Ctsb*, *Gzmb*, *Tnfrsf1a*	*Hras*, *Ptpn13*, *Map3k14*	4.28 × 10^−5^
mmu03030	DNA replication	*Lig1*, *Pold4*	*Mcm2*, *Mcm5*, *Pold1*, *Mcm6*	1.15 × 10^−4^
mmu03040	Spliceosome	*Magohb*, *Slu7*, *Ddx5*, *Prpf40a*	*U2af2*, *Srsf7*, *Hnrnpc*, *Hspa8*	1.68 × 10^−4^
mmu03008	Ribosome biogenesis in eukaryotes	*Riok2*, *Riok1*	*Heatr1*, *Nat10*, *Emg1*, *Rcl1*	3.15 × 10^−4^
mmu04110	Cell cycle	*Cdkn2a*, *Ep300*, *Tgfb2*	*Mcm2*, *Mcm5*	3.62 × 10^−4^
mmu01230	Biosynthesis of amino acids	*Idh1*, *Eno2*	*Mcm4*, *Mcm6*	4.36 × 10^−4^
mmu00531	Glycosaminoglycan degradation	*Hexb*, *Sgsh*	*GusbHyal2*, *Hyal3*, *Idua*	5.37 × 10^−4^
mmu04142	Lysosome	*Ctsd*, *Ctsb*, *Ctsl*	*Hyal2*, *Hyal3*, *Idua*, *Gusb*	7.53 × 10^−4^

**Table 6:** Comparison of the DEGs significantly enriched in KEGG pathways for 1 d and 2 d of induced differentiation.

KWGG-ID	KEGG Pathway	UP-Genes	*p*-Value
mmu05205	Proteoglycans in cancer	*Cdkn1a*, *Fgf2*	1.21 × 10^−14^
mmu04510	Focal adhesion	*Src*, *Thbs1*, *Itga5*	2.21 × 10^−13^
mmu04360	Axon guidance	*Met*, *Ptk2*, *Rhoa*	1.46 × 10^−10^
mmu05418	Fluid shear stress and atherosclerosis	*Egfr*, *Pdgfa*	4.24 × 10^−10^
mmu04151	PI3K-Akt signaling pathway	*Akt3*, *Jak1*, *Osm*	1.90 × 10^−9^
mmu05206	MicroRNAs in cancer	*Vegfa*, *Cdkn1a*	6.37 × 10^−9^
mmu04015	Rap1 signaling pathway	*Itgb1*, *Igf1r*, *Pik3cb*, *Rap1b*	1.59 × 10^−8^
mmu04010	MAPK signaling pathway	*Map2k1*, *Kras*, *Pak1*	3.44 × 10^−8^
mmu04390	Hippo signaling pathway	*Ctnnb1*, *Ccnd1*	4.35 × 10^−8^

## Data Availability

The datasets used and/or analyzed during the current study are available from the corresponding author upon reasonable request.
